# Fungi enhance microbial carbon retention in high Arctic fjord sediment

**DOI:** 10.1371/journal.pbio.3003783

**Published:** 2026-06-16

**Authors:** Juan Carlos Trejos-Espeleta, James A. Bradley, Ömer K. Coskun, Laura M. Wehrmann, Gonzalo V. Gomez-Saez, William D. Orsi

**Affiliations:** 1 Department of Earth and Environmental Sciences, Palaeontology and Geobiology, Ludwig-Maximilians-Universität München, Munich, Germany; 2 Aix Marseille University, University of Toulon, Centre national de la recherche scientifique (CNRS), Institut de Recherche pour le Développement (IRD), Mediterranean Institute of Oceanography (MIO), Marseille, France; 3 School of Biological and Behavioural Sciences, Queen Mary University of London, London, United Kingdom; 4 School of Marine and Atmospheric Sciences, Stony Brook University, Stony Brook, New York, United States of America; 5 GeoBio-Center, Ludwig-Maximilians-Universität München, Munich, Germany; Trent University, CANADA

## Abstract

Fungi serve as critical biological carbon storage reservoirs in soil ecosystems, but whether this fungal trait is also important for marine sediment carbon storage processes is poorly understood. Here, we quantify for the first time assimilation of dissolved free amino acids by fungi in marine sediments from a high Arctic fjord and show that a distinct community of marine fungi promoted the stabilization of assimilated carbon via a relatively high metabolic efficiency. This corresponded to higher in situ ratios of fungi:prokaryote biomass in the fjord benthos, indicating efficient fungal metabolism promotes increased retention of microbial biomass at the seafloor. Quantitative stable isotope probing linked this efficient assimilation of amino acids to more than 80 fungal taxa in the fjord sediments, primarily associated with aquatic hyphomycetes. An efficient assimilation of amino acids is shown here to be a trait of marine fungi that plays a role in retaining labile dissolved organic matter as microbial biomass in Arctic fjord benthic ecosystems, hotspots for carbon sequestration that are currently experiencing rapid change due to climate warming. Our results indicate that fungal metabolism and biomass in marine sediment should be considered as an important contributor to seafloor carbon storage.

## Abstract 2

Fungi act as critical biological carbon storage reservoirs in soil ecosystems, but what about marine sediments? This study shows that marine fungi promote carbon storage in arctic fjord sediment, hot-spots of carbon sequestration that are currently experiencing rapid change due to climate warming.

## Introduction

Arctic fjords are hotspots for carbon sequestration making them an important ecosystem that contributes to climate regulation [1–3]. Global warming is causing temperatures to rise four times faster in the Arctic than the global average [4], which is leading to accelerated glacial melting and caused profound changes in Arctic fjord ecosystems including carbon cycling [5]. High marine primary productivity [6], an abundance of mineral surfaces for organic carbon (OC) sorption [7], and the delivery and accumulation of older and less-reactive terrestrial C to the marine system [8] all contribute to the diversity of OC in Arctic fjords. The role of microbial communities for fjord carbon cycling under these rapidly changing conditions is poorly understood.

The fate of the labile dissolved organic matter (DOM) is crucial to understand, because labile DOM is quickly assimilated by microbes and either converted to particulate organic matter (POM) as microbial biomass, controlling the retention of OC or remineralization as CO_2_ [1]. The efficiency of microbial assimilation of DOM into POM, and respiration as CO_2_, is now recognized as one of the most important regulators of soil carbon cycling [2]. The role of microbial DOM assimilation efficiency in fjord ecosystems is poorly understood by comparison. This is important to understand, because seafloor microbial activity determines how much of the marine OC is remineralized at the fjord sediment surface before it is rapidly buried and sequestered below the seafloor [1,3,9–11].

Marine fungi are ubiquitous throughout the world’s oceans and are hypothesized to make key contributions to carbon and nutrient cycling in marine ecosystems [12,13]. This is underpinned by the enormous global biomass of marine Fungi, which is estimated to be 0.32 Gt C [14]. In the marine environment, fungi can exhibit OC assimilation rates comparable to those of bacteria and archaea [15], and can degrade marine OC such as algal glycans [16,17]. Marine fungi can also be an important source of marine OC, specifically the fungal-specific glycan alpha- 1,6-mannan [18]. These recent findings show that fungi have the potential to play important, but as-of-yet poorly understood roles, in the consumption and production of marine OC. Moreover, marine fungi exhibit high genomic complexity and species diversity, suggesting unique traits relative to bacteria and archaea with potential to influence the global marine carbon cycle [19–21]. Still, an understanding of the diverse ecological roles of fungi in marine environments is only beginning to emerge [22,23].

Fungal parasitism of algae is a common lifestyle in Arctic marine ecosystems [24–27], and freshwater habitats [28,29]. Free living (non-parasitic) fungi also inhabit in Arctic marine ecosystems also in Svalbard [30–32]. Compared to fungal parasites, however, much less attention has been paid to the activity of free-living marine fungi and their role in DOM assimilation in Arctic marine ecosystems. The metabolism and enzymatic potential of fungi in polar versus non-polar marine waters is different, indicating that marine fungi in polar marine ecosystems play a unique role in carbon cycling [33]. Bacterial assimilation and cycling of DOM, including free amino acids (FAAs), is key component of the marine C cycle [34–36]. A similar role of FAAs assimilation and remineralization by free-living osmotrophic fungi in the marine OC cycle is yet to be constrained.

**Fig 1:**
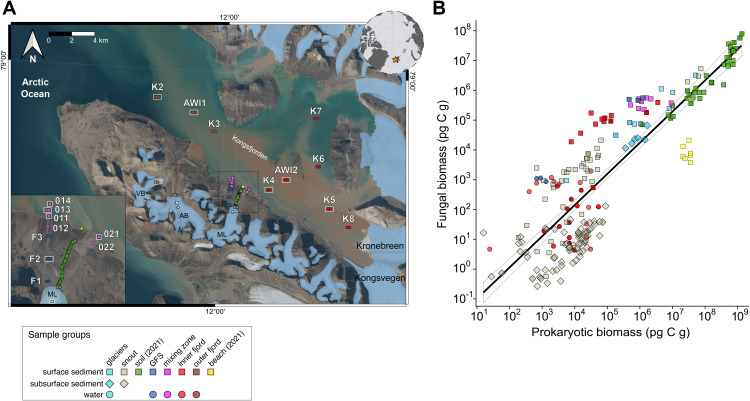
Distribution of sampling sites and microbial abundance. **(A)** Map of the sampling area in the Kongsfjorden and its catchment area (ML, Midtre Lovénbreen; AB, Austre Brøggerbreen; VB, Vestre Brøggerbreen). Source: Norwegian Polar Institute, accessed in January 2025 (https://geodata.npolar.no/arcgis/rest/services/Basisdata/NP_Satellitt_Svalbard_WMTS_25833/MapServer/). Orange star in the map in the upper right corner shows the location of Kongsfjorden in Svalbard within the high Arctic circle. White boxes surrounding symbols indicate sites for SIP incubations. Inset in lower left shows a zoomed in map of sample sites at the land-sea interface. Green squares: proglacial soil chronosequence sampled in 2021 [37]. Symbols indicate sample types: circles: water, squares: surface sediments, diamonds: subsurface sediments. **(B)** A log-log correlation between the fungal and prokaryotic biomass C, which was converted from fungal 18S and prokaryotic 16S rRNA gene abundance using established conversion factors [38–40] (see S1 Fig). The colors and symbols in the legend correspond to those in the map in panel A. GFS, glacier-fed streams. Linear model (log-log): log_10_(Fungi)= -2.04 + 1.05 * log_10_(Prokaryotes).  R_2_ = 0.75, p < 0.001.  Spearman = 0.79, p < 0.001. The data underlying this Figure can be found in S1 Data or at https://doi.org/10.5281/zenodo.19493690.

To better understand the role of fungi in fjord marine carbon transformations, we investigated fungal and bacterial assimilation of FAAs in fjord sediment and seawater in the high Arctic fjord environment of Kongsfjorden, Spitsbergen (Svalbard Archipelago, Norway: between 74° and 81° N) (Fig 1A). FAAs are an important component of labile DOM, and account for 1%–3% of marine dissolved organic carbon (DOC) globally [41]. The remaining 97% of DOC is defined as recalcitrant/refractory [42,43]. FAAs do not accumulate to high levels in the ocean because of rapid uptake by bacteria [44] and in Kongsfjorden (our study area) FAAs are an important labile component of the DOM pool for microbial communities [45]. FAAs are commonly used as model labile DOM compounds in studies of marine microbial carbon cycling [35,36,46].

Arctic fjords receive a diversity of OC compounds that range in reactivity [47]. Microbial assimilation or remineralization of labile DOM, including FAAs, at the sediment surface helps determine how much OC is stored in the fjord sediment [3,9]. It is therefore important to better understand, how microbes retain and or stabilize of labile DOM as biomass in Arctic fjord sediments [46,48,49]. qSIP measures quantitative assimilation of a labeled substrate by specific bacterial [50–52] or fungal [15,50,53,37] taxa in complex natural microbial communities. Here, we conducted quantitative DNA stable isotope probing (qSIP) experiments using ^13^C-, ^15^N-labeled FAAs to quantify assimilation of labile DOM by fungi and bacteria, separately. The qSIP results were used to assess the ecological role of marine fungi for labile carbon retention in Kongsfjorden, a sentinel marine ecosystem [54,55] in the rapidly changing high-Arctic.

## Results

### High fungi:prokaryote biomass ratios in Arctic fjord sediment

During the summers of 2023 and 2024, we sampled a wide range of habitats in Kongsfjorden including sediments and seawater from multiple sites in the fjord, as well as sediments and brackish water from discharge plumes of glacier-fed streams (GFSs) into the fjord. We also sampled upstream environments including GFSs, soils, glacial till, and glacial sediment (Fig 1A). Kongsfjorden hosts several large marine-terminating glaciers at the fjord head (Kronebreen, Kongsvegen, Kongsbreen) and several land-terminating smaller glaciers, that deliver runoff by glacial streams (Fig 1A). In total, we collected 240 sediment and water samples from 64 different locations, ranging from the glacier surface to the fjord seafloor.

Quantitative PCR (qPCR) of fungal 18S ribosomal RNA (rRNA) and 16S rRNA (targeting bacteria and archaea) were used to measure fungal and prokaryote rRNA gene abundances. The qPCR amplification of fungal-specific 18S rRNA genes are derived primarily from fungi, particularly in the marine samples, because blocking primers [56] were used to reduce amplification of 18S rRNA genes from protists and other non-fungal eukaryotes. The rRNA gene abundances were converted to biomass estimates using conversion factors previously established to convert fungal [38,39] and prokaryotic [40] gene copy concentrations to biomass (see Methods). A comparison of fungal to prokaryotic estimated biomass revealed a positive log–log correlation (Pearson: *R*^2^ = 0.824, *p* < 0.001) across all sites sampled (Fig 1B), which mirrored the correlation based on qPCR determined gene copy abundances (S1 Fig). The positive correlation between fungal and prokaryote biomass (Fig 1B) was generally consistent across most marine and terrestrial sites, with the majority of soil and sediment habitats having higher microbial biomass compared to seawater samples (Fig 1B). The highest microbial biomass was observed in organic-rich habitats such as soils, fjord sediment, and GFS sediments, whereas the lowest microbial biomass was found in comparatively organic-lean environments like subsurface glacial till sediments and fjord seawater (Fig 1B).

**Fig 2:**
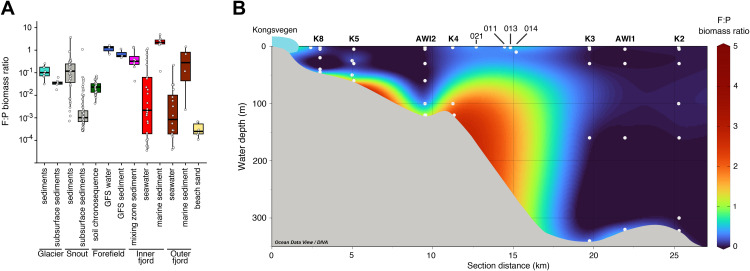
Fungal to prokaryotic biomass ratio across fjord habitats. **(A)** Fungi:prokaryote (F:P) biomass ratios in habitats across the land-sea continuum in the Kongsfjorden and its catchment area. Values are the ratio of fungal and prokaryotic biomass concentrations, after converting from gene copy abundance using established conversion factors [38–40] (S1 Fig) (see Methods). Boxes represent the lower and higher quartile values; the median is a horizontal line. GFS: glacier-fed stream. **(B)** Vertical profile of F:P biomass ratios from sea surface to seafloor across the fjord transect from Kongsvegen glacier (left) until the outermost fjord site (K2). White dots indicate sampling points. Colors are interpolated values between the points were calculated with Ocean Data View [57]. See map in Fig 1 for site locations. The data underlying this Figure can be found in S1 Data or at https://doi.org/10.5281/zenodo.19493690.

Ratios of fungi to prokaryote biomass (F:P biomass ratios) were the highest in the inner fjord sediments, followed by fjord mixing zone sediments and GFS sediment and fjord seawater (Fig 2A). Notably, the median F:P biomass ratios in the inner fjord sediments were three orders of magnitude higher compared to nearby tundra soils (Fig 2A). Within the fjord, the F:P biomass ratios were higher in the inner fjord sediments closest to the marine-terminating glacier and decrease with increasing distance from the glacier towards the open ocean in the outer fjord sediments (Fig 2B). In contrast to sediments, median F:P biomass ratios in seawater were several orders of magnitude lower (Fig 2A). The lowest median F:P biomass ratio was found in beach sand, which was two to four orders of magnitude lower than the median F:P biomass ratio in the fjord sediments (Fig 2A).

**Fig 3:**
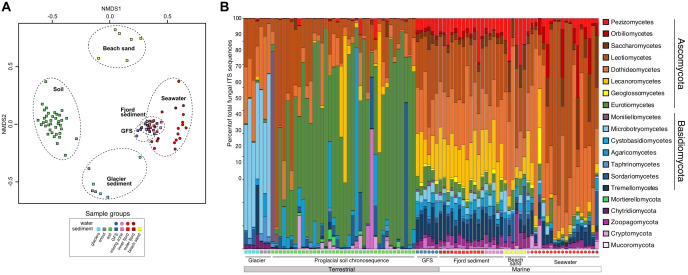
Fungal community composition in the fjord differs from other environments. **(A)** Non-metric multidimensional scaling (NMDS) analysis of habitat groups (S1 Table) based on fungal ITS-defined OTU distributions. **(B)** Relative abundance of major fungal groups based on ITS barcoding, across the land-ocean continuum in the Kongsfjorden catchment area (see map in Fig 1). GFS: glacier-fed stream. The data underlying this Figure can be found in S1 Data or at https://doi.org/10.5281/zenodo.19493690.

Next, we profiled the diversity of fungi across Kongsfjorden via high-throughput sequencing of the internal transcribed spacer (ITS) region in order to assess which fungal groups were responsible for the higher F:P biomass ratios in the fjord sediments and GFSs. Fungal community composition was different between the glacial, soil, and marine habitats (Analysis of Similarity; *R* = 0.7, *P* = 0.001; Fig 3A), which was driven largely due to a higher relative abundance of the ascomycete fungal groups Pezizomycetes, Orbiliomycetes, Saccharomycetes, Dothideomycetes, and Letiomycetes in the fjord sediments and GFSs compared to other habitats (Fig 3B). Notably, fungal communities in the fjord sediment were more similar to GFSs compared to fungal communities in the overlying seawater (Fig 3A).

### Respiration and OC utilization by fungi and prokaryotes

In the seawater SIP incubations, respiration was relatively slow and O_2_ remained present in the seawater SIP experiments at concentrations >250 µM over the entire incubation (S2 Fig). By comparison, in the sediment incubations respiration consumed O_2_ much faster, and the sediment-water interface of the flasks containing inner and outer fjord sediment became anoxic within two hours after beginning the incubations (S2 Fig).

**Fig 4:**
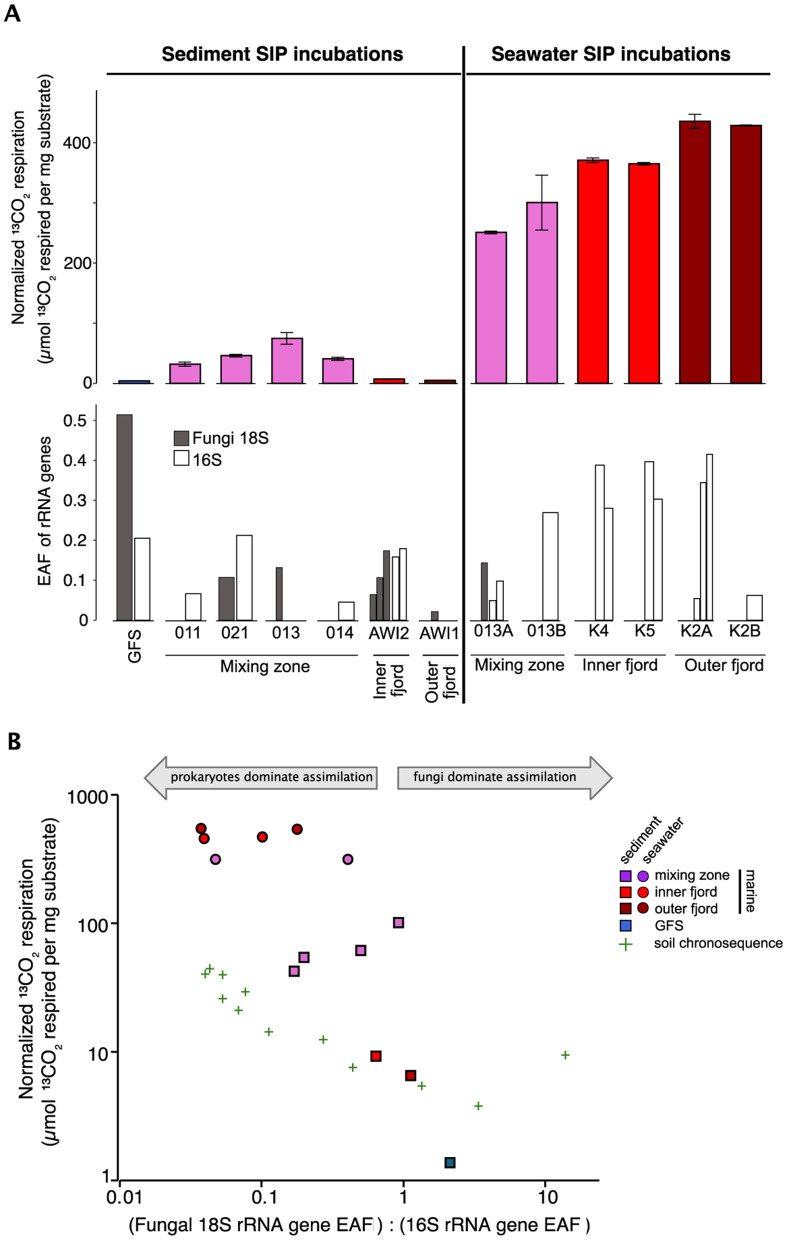
Carbon assimilation and remineralization in sediment and seawater incubations. **(A) Top**: Normalized ^13^CO_2_ production in the SIP incubations. Bar charts and error bars represent average and standard error from three technical replicates, respectively. **Bottom**: Excess atom fraction (EAF) of fungal 18S and prokaryotic 16S rRNA genes. Biological replicates are labeled “A” and “B,” technical replicates are multiple bars within each biological replicate. All seawater SIP incubations were from 2 m water depth. **(B)** Log-log plot between ^13^CO_2_ production and median fungi:prokaryote EAF ratios in the fjord catchment showing a significant negative correlation (Spearman’s rank correlation: rho = −0.79, *P* = 0.001). Biological replicates are plotted individually. For biological replicates that had multiple technical replicates, the median value was taken (see S2 Table). Green crosses represent SIP incubations of forefield soils of Midtre Lovénbreen glacier (Fig 1) published previously [37]. GFS: glacier-fed stream sediment. The data underlying this Figure can be found in S1 Data or at https://doi.org/10.5281/zenodo.19493690.

The normalized FAA ^13^CO_2_ respiration rates from seawater were one to two orders of magnitude higher compared to sediment (S3 Fig). Specifically, fjord and GFS sediments had the lowest remineralization rates (Fig 4A). From the SIP incubations, DNA buoyant density shifts in prokaryotic 16S rRNA genes and fungal 18S rRNA genes allowed us to quantify microbial assimilation of ^13^C, ^15^N FAAs and calculate the excess atom fraction (EAF) in 16S and fungal 18S rRNA genes (S4 and S5 Figs). The EAF value serves as an estimate for the percentage of C and N atoms in the rRNA gene that has been replaced with the stable isotopes from the added labeled FAAs substrate. Blocking primers reduced amplification of non-fungal eukaryote 18S rRNA genes [56], and thus the EAF values obtained are indicative of fungal amino acid assimilation specifically. ^13^C-cellulose was respired slower compared to the ^13^C-FAAs, both in the water column and in sediments, at all sites (S3 Fig). Consistent with this, in the ^13^C-cellulose SIP incubations no detectable ^13^C-labeling of fungal 18S rRNA genes or prokaryotic 16S rRNA genes from labeled cellulose was observed (S6 Fig) (EAF < 0.01).

Prokaryotes assimilated FAAs in all incubations except for sediments from site 013 (mixing zone) and site AWI1 (outer fjord), whereas fungal assimilation was detected in five sediment incubations (GFS, 021, 013, AWI2, AWI1) and in one replicate of the seawater SIP incubation from the fjord mixing zone (013A) (Fig 4A). Fungi in the fjord sediments and GFS sediments had on average higher FAA assimilation compared to seawater (Fig 4A). We observed a higher amount of ^13^C,^15^N-FAA assimilation by fungi at the inner fjord site AWI2, compared to the outer site AWI1 (Fig 4A), corresponding with a high oxygen consumption rate that resulted in anoxic conditions at the sediment-water interface (S2 Fig). The ^13^C, ^15^N labeling of fungal 18S rRNA genes was furthermore reproducible across three technical SIP replicates in fjord sediments from site AWI2 (Fig 4A).

In seawater SIP incubations, higher FAA remineralization rates (S3 Fig) and prokaryotic ^13^C,^15^N-EAF values were observed compared to sediments (Fig 4A). This was observed at all sites sampled and across biological replicates. This finding could be due to microbes in the sediments having a preference for other types of naturally existing organic matter, opposed to the added FAAs. In sediments, fungi exhibited ^13^C,^15^N-FAA assimilation in more than half of the sites tested (*n* = 7), and these incubations were associated with lower ^13^CO_2_ respiration rates compared to seawater. In the fjord sediment SIP incubations, fungal 18S rRNA gene EAF was comparable to prokaryotes (and greater than prokaryotes in the case of GFS sediment) (Fig 4A).

### Fungi in fjord sediment have high amino acid assimilation efficiency

In order to assess the relative contributions of prokaryotes and fungi to carbon remineralization and assimilation in the fjord seafloor surface sediment, we compared median fungal:prokaryotic FAA assimilation (F:P EAF ratios) to bulk FAA remineralization (Fig 4B). Across all of the incubations performed in the fjord, the F:P EAF ratio was negatively correlated with ^13^CO_2_ production (Spearman’s rank correlation: rho = −0.79, *P* = 0.001) (Fig 4B). Incubations of surface sediments from the inner fjord (AWI2) site exhibited one of the highest F:P EAF ratios and the lowest ^13^CO_2_ production (Fig 4B). This finding is consistent with relatively high F:P biomass ratios in the inner fjord sediments (Fig 2B). These results prompted us to investigate which fungal taxa were most important for FAA assimilation in the fjord sediment using qSIP.

### Quantifying fungal taxon-specific FAA assimilation with qSIP

To identify the FAA assimilating fungi in the fjord sediment, we performed barcoded ITS sequencing of 10 density fractions across three technical replicates of the control and ^13^C,^15^N-labeled SIP incubations (*n* = 60 total individual density fractions sequenced) from the inner fjord sediment site (AWI2) (S4 Fig). Our use of 10 fractions for amplicon sequencing is in line with the recommendation number of fractions for amplicon sequencing in qSIP experiments for a reasonable tradeoff between precision and cost [58]. The fungal 18S rRNA gene abundances determined via qPCR (S3 Fig) were used to quantitatively normalize the abundance of fungal ITS sequences across the density gradients to calculate taxon-specific shifts in DNA buoyant density according to the qSIP approach described previously for fungi [37]. We acknowledge variation in rRNA gene copies between fungal taxa [59] may bias fungal EAF comparison between different fungal taxa, but this bias should not apply when comparing EAFs from the same fungal OTU between different samples [37].

**Fig 5:**
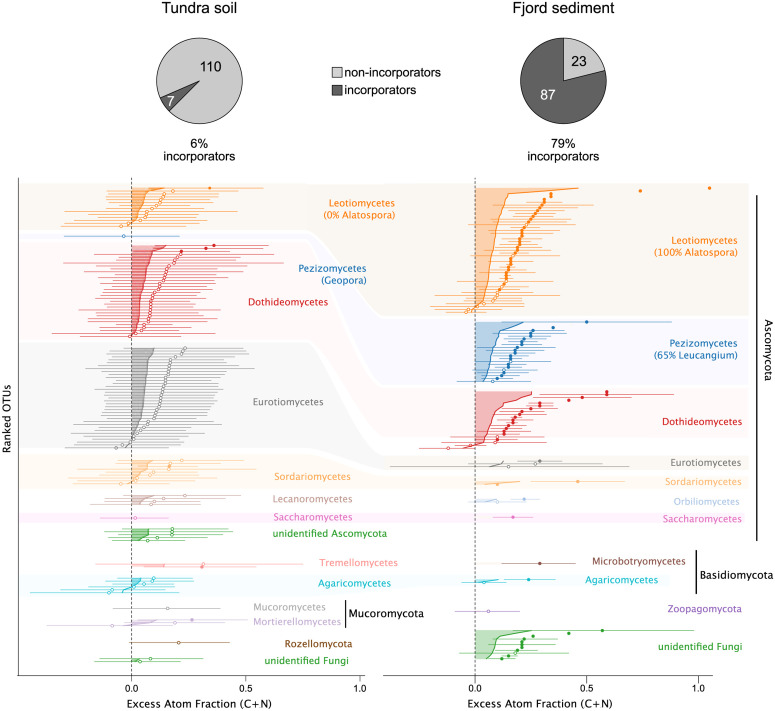
Amino acid assimilating fungal taxa in fjord sediment compared to tundra soil. Excess atomic fraction (^13^C + ^15^N EAF) of fungal OTUs in qSIP experiments with ^13^C,^15^N labeled FAAs in fjord sediment and tundra soil. Points represent bootstrapped ^13^C + ^15^N EAF median values across three technical replicates (EAF = 1.0 is equivalent to 100% of C and N atoms replaced with ^13^C and ^15^N). The shaded area underneath the points represents the estimated amount of EAF due to ^15^N, each point is the estimated amount of EAF due to ^13^C + ^15^N from the labeled FAAs. Error bars correspond to 90% confidence intervals of the EAF medians across technical replicates (*n* = 3). Filled circles: labeled OTUs, unfilled circles: unlabeled OTUs. Colored regions connect taxonomic groups across the two qSIP experiments, but does not imply a continuity between sites. Pie charts show the proportion of labeled OTUs (incorporators) and unlabeled OTUs (non-incorporators) in each location. The data underlying this Figure can be found in S1 Data or at https://doi.org/10.5281/zenodo.19493690.

The qSIP experiment from the fjord sediment identified 87 fungal operational taxonomic units (OTUs) that assimilated ^13^C,^15^N-FAAs (Fig 5). We compared these results to existing fungal qSIP results from nearby tundra soils in the Kongsfjorden catchment area, that was carried out in 2021 (Fig 5) [37]. The fjord sediment and tundra soil qSIP experiments underwent identical incubation conditions (see Methods), and therefore permit a direct comparison of FAA-assimilating fungal taxa between the two environments (Fig 5). The comparison shows that there was a higher number of fungal FAA-assimilating OTUs in the fjord sediments (*n* = 87) compared to nearby soils (*n* = 7) (Fig 5).

The majority (90%) of fungal taxa assimilating FAAs in the fjord sediment (*n* = 87) did not assimilate FAAs in the tundra soil (Fig 5). These corresponded mostly to numerous OTUs within the ascomycete classes Leotiomycetes, Orbiliomycetes, and Pezizomycetes (Fig 5). Fungal taxa that only assimilated FAAs in the fjord sediment (and not in tundra soil) were affiliated with the following genera: *Alatospora* (Letiomycetes) (*n* = 34 OTUs, average EAF = 0.25 ± 0.03), *Orbilia* (Orbiliomycetes) (*n* = 1 OTU, EAF = 0.22), *Patellaria* and *Mycoleptodiscus* (Dothideomycetes) (*n* = 10 OTUs, average EAF = 0.24 ± 0.04), *Leucangium* and *Microstoma* (Pezizomycetes) (*n* = 15 OTUs, average EAF = 0.23 ± 0.03) (Fig 5). All fungal Leotiomycetes OTUs that assimilated FAAs in the fjord sediment (*n* = 34) were affiliated with the genus *Alatospora* (Fig 5).

To better understand the biogeography of active fungi in the fjord sediment, we investigated the distribution of fungal OTUs from genera exhibiting FAA assimilation only in the fjord sediment. This included fungal OTUs affiliated with *Alatospora*, *Mycoleptodiscus*, *Patellaria*, *Orbilia*, *Leucangium*, *Microstoma*, and *Basidiobolous* (Fig 5). The relative abundance of these fungal genera that assimilated FAAs primarily in the fjord sediments was significantly higher (S7 Fig, two-sided *t* test: *P* = 0.003 × 10^−19^) in the fjord ecosystem and GFSs—compared to nearby tundra soil [37] (S7 Fig). Despite the close proximity to the fjord (Fig 1A), most fungal OTUs assimilating FAAs in tundra soils were not active in fjord sediments (Figs 5 and S7).

## Discussion

The role of fungi in the marine carbon cycle is poorly understood [22,28]. Carbon assimilation is a trait of marine fungi that is potentially important [15] considering that carbon assimilation by terrestrial fungi controls soil carbon and nutrient stabilization in soils [60]. Globally, marine fungi are now known to have a significant contribution to marine biomass estimated at 0.32 Gt C [14], which further underscores their likely important role in the marine carbon cycle. Here, we show for the first time that DOM assimilation efficiency of marine fungi living in Arctic fjord sediments is relatively high compared to prokaryotes and that this enhances retention of labile DOM as fungal biomass.

Consistent with previous reports from our study area [24,30,61], we found distinct communities of fungi across the land-sea interface that were significantly different between the tundra, glacial, and marine fjord ecosystems (Fig 3A). The distinct fungal communities in the marine sediment habitats sampled in the fjord coincides with higher F:P biomass ratios (Fig 1B). The lower biomass of fungi in seawater compared to sediment, is likely due to many fungi being adapted to grow in environments that are rich in nutrients and OC [22], such as marine sediment. The strong correlation of fungal and prokaryotic biomass across all marine (fjord) and terrestrial habitats in the catchment area (Fig 1B) indicate that the abundance of both bacteria and fungi are both controlled to a large extent by the availability of organic matter and or nutrients essential for growth. The fungal biomass that we estimated from the gene copy concentrations from soils, marine sediments, and seawater is within the same orders of magnitude compared to similar habitats [14,38,39]. Namely, fjord sediment fungal biomass ranged from 10^4^ to 10^6^ pg C g, spanning the same concentration range as previously reported for deep-sea sediment fungal biomass [38]. In fjord seawater, the fungal biomass ranged from 10^2^ to 10^4^ pg C g (S1 Fig), which covers the range of fungal biomass concentrations reported previously [14]. Although the conversion from gene copy estimates to biomass C is biased by variation in gene copy numbers per cell, the conversions we obtained for fungal biomass C were comparable to prior studies from similar habitats [14,38,39].

In the fjord sediments, the higher F:P biomass ratio in fjord sediments coincides with roughly 10-fold higher sediment OC concentrations compared to seawater [6,62]. This indicates that the increased fungal biomass enhances carbon storage potential in sediment, similar to the enhancement in soils [63]. This finding is consistent with the hypothesis that marine fungi are more active in nutrient-rich environments [22]. Overall, our finding of increased F:P biomass ratios in the fjord sediments (Fig 2A) indicates the fjord benthos as a hotspot for fungal activity, which we could link to specific fungal taxa in the qSIP experiments.

In any SIP experiment, the concentration of naturally existing substrates that microbes are exposed to, in relation to the concentration of stable isotope-labeled substrates provided, will impact the results, and so it is important to place them into context. Dissolved organic nitrogen (DON) concentrations in Kongsfjorden range from 2 to 25 µM [6,62]. Plant-derived OC, including cellulose, represents 2% of Svalbard fjord sediment total OC, respectively [11]. Sediment DOC concentrations are roughly 100-fold higher compared to seawater in Kongsfjorden [6,62]. To reflect the naturally existing two-order-of-magnitude difference in DOC concentrations between seawater and sediment, we added the FAAs at 100-fold higher concentrations in the sediment SIP incubations (10 µg g^−1^) compared to seawater SIP incubations (0.1 µg mL^−1^). Based on prior measurements [6,62], we estimate the ^13^C enrichment of DOC in the seawater SIP incubations should have been roughly 3%–6% whereas the ^13^C enrichment of DOC in the sediment SIP incubations was roughly 14%–16% (see Methods). The higher ^13^C enrichment of DOC in the sediment SIP incubations may have influenced sediment microbial activity and selected for more copiotrophic microbes, including fungi.

In addition to the constraints imposed by the substrate concentrations, the dual labeled ^13^C^15^N FAA substrate approach that we used makes it challenging to parse out assimilation of carbon versus utilization of nitrogen from the FAAs. For example, in nutrient-limited conditions bacteria and or fungi could respire the FAAs carbon as CO_2_, and assimilate the nitrogen from ammonia after FAA deamination (for biosynthesis). Such metabolic choices can be taxon-specific [64], and therefore parsing out taxon-specific use of ^13^C and or ^15^N would require additional, separate experiments with a single isotopic label. We therefore interpret the qSIP results of fungi as those taxa that were assimilating amino acids, and refrain from speculation regarding taxon-specific assimilation preferences of amino acid C or N.

Rates of respiration and assimilation in SIP experiments are impacted by the concentration of the added substrate [65]. The seawater and sediments investigated here are located in a fjord ecosystem that is hydrologically dynamic and experiences large seasonal fluctuations in primary productivity [54]. Thus, the addition of FAA substrates in the SIP incubations was reflective of variation in OC availability that the microbes are naturally exposed to. Nevertheless, even though we added the FAAs at concentrations <16% of the natural DOC in Kongsfjorden [6,62] (see Methods), the addition of FAAs may have increased microbial activity and increased rates in the SIP incubations relative to the in situ state at the time of sampling. We assess the relative assimilation rates to compare how FAAs are utilized by fungi and do not attempt to quantify absolute in situ carbon fluxes.

In seawater SIP incubations prokaryotes dominated FAA assimilation over fungi, whereas fungi assimilated more FAAs in the sediment incubations under higher substrate concentrations (Fig 4A). The higher FAA assimilation of seawater prokaryotes compared to fungi could be related to higher amino acid uptake affinity of prokaryotes compared to fungi [65] and or growth of planktonic marine fungi in seawater being limited by lower concentrations of organic matter [22]. Although prokaryotes dominated FAA assimilation over fungi in fjord seawater, it was relatively inefficient and resulted in increased CO_2_ production compared to benthic SIP incubations (Fig 4B). The increased fungal assimilation of FAAs in sediments, coupled with the lower rates of FAA remineralization, shows that FAA-assimilating fungi in the sediment had an increased efficiency of DOM assimilation. This in turn, contribute to increased retention of labile OC retention as fungal biomass and can potentially explain the higher fungal:prokarytoic biomass ratios in the fjord sediments (Fig 2).

Our results indicate that the balance between fungal carbon assimilation and respiration is a key parameter controlling stabilization of labile DOM as biomass in the fjord sediments. Consequently, fungal FAA assimilation efficiency in fjord sediments should result in an increased OC retention as microbial biomass in fjord sediment. This should be considered as contributing to the overall microbial OC assimilation that is is ultimately buried and sequestered as biomass below the seafloor [46]. This process should be influenced by the sedimentation rate that controls how fast the DOM assimilating microbes are buried. If a substantial portion of newly formed fungal biomass becomes buried below the seafloor during sedimentation, the fungal carbon retention we observed in our experiments may even contribute to a longer-term carbon sink (e.g., on geological timescales). This possibility is worth investigating in the future, considering the extremely fast sedimentation rates in fjords [1,3,9].

The higher F:P biomass ratios in the fjord sediment coinciding with increased fungal FAA assimilation efficiency, is consistent with terrestrial ecosystems where increased F:P biomass ratios also correlate with increased carbon storage [37,63]. We compared qSIP experiments from nearby tundra soil [37] to the fjord qSIP experiments presented here, which shows that in both the tundra soil and fjord sediments the F:P EAF ratios have an inverse correlation with ^13^CO_2_ production (Fig 4B). Collectively, these results show that fungal DOM assimilation efficiency is a trait helping to retain labile DOM as fungal biomass in Arctic fjord sediment.

Identification of the key fungal taxa for FAA assimilation in the fjord sediment revealed a more than 10-fold higher number of fungal OTUs assimilating FAAs compared to tundra soil (Fig 5). Within the fjord sediment, numerous FAA-assimilating OTUs were affiliated with the genus *Alatospora* (Fig 5). *Alatospora* is a genus of aquatic hyphomycete fungi, a polyphyletic group with at least 300 different species [66,67] that are known to be important for leaf litter degradation in streams [67,68]. The geographic distribution of *Alatospora* is globally widespread, and has been found to extend to the sub-Arctic [69]. Our results reveal an ecological role for marine *Alatospora* species as important FAA-assimilating fungi in Arctic fjord sediment. In addition to *Alatospora*, there were also a relatively high number of FAA assimilating fungal OTUs affiliated with *Patellaria* and *Mycoleptodiscus*—genera that frequently occur in mangroves [70]. For example, *Patellaria* is a saprotrophic fungus that can colonize wood and is often distributed in marine environments [71,72]. Our results suggest that fungal taxa within *Patellaria* and *Mycoleptodiscus* are important for labile DOM retention in Arctic marine sediments. The relatively high number of Pezizomycetes-affiliated OTUs that assimilated FAAs exclusively in the fjord sediment (Fig 5) is consistent with prior findings that Pezizomycotina fungi have been detected ubiquitously across Arctic marine ecosystems [26]. This is consistent with our survey of fungal ITS diversity across the catchment area, where Pezizomycetes-affiliated fungi were relatively abundant in the marine environment (Fig 3B).

Our qSIP experiments show that diverse fungal taxa within the Pezizomycotina, *Alatospora*, *Patellaria*, and *Mycoleptodiscus* taxa were primarily responsible for the FAA assimilation (Fig 4B) and retention as fungal biomass in the fjord sediment. These active marine fungal taxa in fjord sediments were specific to the fjord environment, and were not observed to be a major component of the terrestrial fungal community (S7 Fig). This points to a distinct benthic Arctic marine fungal community that is enriched with fungal populations adapted to assimilate labile DOM.

The sediments of the Arctic fjord are anoxic 0.3–0.8 cm below the surface [10]. We attempted to maintain similar conditions in the qSIP incubations from fjord sediments, and established anoxic conditions at the sediment-water interface (S2 Fig). Therefore, the *Alatospora* assimilating amino acids in the qSIP experiments of AWI2 fjord sediments (Fig 5) were able to maintain this activity even under anoxic conditions. However, the flasks were open to the air, and therefore oxygen was diffusing into the surface of the seawater ca. 2 cm above the sediment likely forming a redox gradient and oxic-anoxic interface above the sediment. It is therefore possible that the active fungi living in the water above the sediment still had access to low amounts of O_2_ diffusing in from above. Considering the anoxic conditions at the sediment surface however, it appears that the diverse *Alatospora* taxa, and many other marine fungi (Fig 5), can maintain relatively high metabolic activity even under low-oxygen, and or anoxic, conditions.

The activity of *Alatospora* fungi under anoxic conditions could be explained by a facultatively anaerobic metabolism. Namely, it is possible that some species of *Alatospora* can maintain high metabolic activity under anoxic conditions via fermentation, or possibly even denitrification. Denitrification to our knowledge has never been shown in *Alatospora*, but it has been demonstrated for several marine fungi and is an important trait in low-oxygen marine environments [19]. Our findings show that the anaerobic metabolism of *Alatospora* in marine sediments, as relates to carbon cycling and storage, requires future study for a full characterization.

Fjord sediments are critical regulators of carbon storage with microbial communities that control carbon stabilization at the fjord seafloor surface prior to burial [55]. The role of fungi in this process is poorly understood. In this study, we show that fungi living at the seafloor in a high Arctic glacial fjord ecosystem have a relatively efficient FAA assimilation, stabilizing this important labile component of marine DOM as biomass. This could explain the observation of higher ratios of fungal:prokaryote biomass in fjord sediment, and indicates that labile DOM is an important substrate for marine fungi living in the benthos of high Arctic fjords. Considering the significant global contribution of marine fungal biomass to OC in seawater [14], seafloor fungi with efficient DOM assimilation in marine sediment may represent an additional major reservoir of marine OC.

## Methods

### Study sites and sampling

Sediment, seawater, and soil samples were collected in early August 2023 and late July 2024, in the area surrounding Kongsfjorden and the Brøggerhalvøya peninsula in Svalbard (12.0° E, 79.0° N, Fig 1). Additionally, the Midtre Lovénbreen soil chronosequence was sampled in 2021 [37]. In Kongsfjorden, summer conditions can differ from year to year—but the fjord is generally no longer frozen over with ice starting from late April (during which the spring phytoplankton bloom usually occurs) until October [69]. Sampling in multiple years was required in order to complete the sampling of a large region from many different environments in the catchment area (fjord sediment, seawater, glaciers, soil, streams, etc) that is located in a remote area of the high Arctic. We sampled from the following land-terminating glaciers and their respective forefields: Midtre Lovénbreen (ML), Austre Brøggerbreen (AB), and Vestre Brøggerbreen (VB). We categorized sample sites into the following groups and sub-groups according to the location and nature of the sample (Fig 1A and S1 Table): glacier’s water, surface and subsurface sediment, snout’s surface and subsurface sediment, glacial forefield soil chronosequence, glacier-fed stream’s water and sediment (GFS), mixing zone’s water and sediment, inner fjord’s seawater and sediment, and outer fjord’s seawater and sediment (Fig 1 and S1 Table). The mixing zone is defined here as the area where the GFS reach the fjord, and exhibits particle-rich plumes of brackish water (Fig 1). The fjord water column is vertically stratified [54,73] and regularly experiences an annual spring phytoplankton bloom beginning in late April [74]. Discharge plumes of brackish water with high loads of suspended particulate matter develop in the fjord adjacent to marine-terminating glaciers [75] (Fig 1A).

Fjord sediment samples were collected in 2023 at sites AWI1 and AWI2 (Fig 1A) aboard the MS Teisten (Kings Bay AS) using a sediment multi-corer. Retrieved cores were brought back to land within 4–6 h and subsampled immediately. Surface sediment from the core-top was used to set up the fjord sediment qSIP experiment. Additional fjord sediment samples were collected in 2024 aboard the MS Teisten (Kings Bay AS), using a van Veen grab sediment sampler at fjord sites: K8, K7, K6, K5, K4, K3, K2, AWI2, and AWI1 (Fig 1A). This material was frozen in sterile 2 mL cryotubes and used for DNA extraction, qPCR, and fungal ITS1 barcoding. Surface seawater (2 m depth) was sampled from the fjord using compressible plastic bottles attached to a rope. Deeper seawater samples in the water column were sampled using a 10 L Niskin bottle onboard the MS Teisten (Kings Bay AS), brought back to land within 4 hours, and either filtered onto 0.2 µm filters immediately for DNA extraction or used for SIP incubations (see below). Mixing zone surface sediments were collected manually with a 15-mL Falcon tube at <2 m water depth sites (011, 012, 013, 021, 022; Fig 1A). Surface sediments from the glacier and snout were collected directly with a 15 mL Falcon tube or with a sterilized spatula into 2 mL sealed microcentrifuge tubes. Water samples were collected from GFS using a 500 mL syringe and were filtered on site onto hydrophilic polyethersulfone (PES) filters (0.2 μm pore diameter). Seawater was filtered using a Masterflex L/S peristaltic pump (Model 07528-30, Cole-Parmer) with precision pump tubing (PharMed BPT tubing, Cole-Parmer) connected to an inline filter holder containing 0.2 μm PES filters. Filters were kept inside 2 mL screw-cap sealed tubes and frozen at −20 °C. All sediment samples and filters were kept at −20 °C until DNA extraction.

### SIP incubation setup

We added different concentrations of substrates (amino acids and cellulose) to sediment and seawater SIP incubations to account for the large difference in DOC concentrations between these habitats (as has been previously reported for surface sediment porewater and seawater in Kongsfjorden [6,62]). Specifically, the concentration of DOC in Kongsfjorden sediment ranges from 10,000–12,000 µM compared to DOC concentrations in seawater that range from 10 to 200 µM [6,62]. Therefore, we added labeled or unlabeled substrates at 100-fold higher concentrations in the sediment SIP incubations (10 µg mL^−1^) compared to seawater SIP incubations (0.1 µg mL^−1^).

Assuming the seawater DOC is between 100 and 200 µM [6,62] the ^13^C enrichment of seawater DOC in the SIP incubations should have been roughly 3%–6%. In contrast, assuming the sediment DOC is between 10,000 and 12,000 µM [6,62] the ^13^C enrichment of the sediment SIP incubations should have been roughly 14%–16%. The reason for the higher enrichment in the sediment SIP incubations is the mixture of sediment (3 mL) and bottom seawater (7 mL), which results in a dilution of the sediment DOC concentration (because the seawater DOC is 100-fold less concentrated). Considering this together with the 100-fold higher labeled amino acid substrates, the final ^13^C-enrichment of the DOC in the sediment SIP incubations should have been higher compared to the seawater SIP incubations.

Adding the labeled amino acid substrates at these relatively low concentrations, should reduce the risk of “tipping the system” away from native microbial activities, but to nevertheless provides enough organic substrate to achieve detectable EAF values in 16S and fungal 18S rRNA genes [37]. Our chosen concentration is within the general concentration range considered to be required for detecting DNA labeling with high-resolution DNA-SIP, and is similar to the concentrations of ^13^C-labeled amino acids used by previous DNA-SIP studies to trace bacterial and fungal carbon assimilation [37]. All SIP incubations were performed in the dark at 4 °C for seven days, and were set up within 24 hours of obtaining the samples. Samples were incubated in triplicates to assess biological variability and some samples were analyzed in technical replicates to assess analytical variability.

We used commercial amino acid mixtures for SIP incubations: ^13^C,^15^N-labeled (Sigma-Aldrich, Cat# 767964-1EA) and unlabeled (Sigma-Aldrich, Cat# 79248-5X2ML) as a control. The mixtures consisted of 17 amino acids naturally found as FAAs (alanine, arginine, aspartic acid, glutamic acid, glycine, histidine, isoleucine, leucine, lysine, methionine, phenylalanine, proline, serine, threonine, tyrosine, valine, and cystine). For tracing cellulose utilization in SIP incubations, ^13^C-labeled cellulose (Sigma-Aldrich, Cat# 696498) was used. We applied unlabeled cellulose (Sigma-Aldrich, Cat# 310697) as a control. Sediment incubations were performed in 10 mL borosilicate vials by adding 3 mL of wet sediment (5 g) with a cutoff sterile syringe, followed by labeled or unlabeled substrate (either amino acids or cellulose) to the sediments. The sediment was then mixed by drawing material in and out and stirring with a pipette. In sediment SIP incubations, bottom seawater from the corresponding sample site was added to fill the headspace of the 10 mL vial before covering with parafilm. Seawater SIP incubations were performed using 1 L of seawater, and prepared in 1 L borosilicate bottles with the plastic screw cap slightly loose permitting the exchange of air and prevent oxygen depleted (or potentially anoxic) conditions from developing in seawater incubations. We acknowledge that by loosening the caps to allow oxygen exchange, some ^13^CO_2_ could have been lost during the incubation and may have impacted the remineralization rate measurements. However, because the pH of the seawater and sediments is slightly alkaline (e.g., between 7.8 and 7.9)—most of the inorganic carbon (>90%) is in the form of bicarbonate (not CO_2_) which would have retained the majority of remineralized carbon as bicarbonate in the dissolved inorganic carbon (DIC) pool.

Sediment SIP incubations were terminated by immediately freezing the sediments in their glass vials at −20 °C. Seawater SIP incubations were processed by filtering the seawater using a peristaltic pump (Masterflex L/S peristaltic pump, Cole-Parmer) with precision pump tubing (PharMed BPT tubing, Cole-Parmer) connected to an inline filter holder containing a 0.2 μm PES filter. The filters were frozen immediately at −20 °C. During filtration, 15 mL of 0.2 µm seawater filtrate was collected and frozen for gas chromatography quadrupole mass spectrometry (GC–QMS) measurements of the remineralized ^13^CO_2_.

### Dissolved O_2_ measurements

During the SIP incubations, we monitored the consumption of O_2_ inside the flasks or bottles with a non-invasive method as described previously [15]. In brief, prior to setting up the experiment oxygen sensor spots (PreSens) were glued to the inside of the glass flasks using a silicon glue and dried overnight. Oxygen sensor spots were then sterilized with 70% ethanol prior to setting up the incubations. For the sediment incubations, sensor spots were glued to a position in the flasks such that they were located at the sediment–water interface inside the glass flasks. For the seawater incubations, oxygen sensor spots were positions ca. 3 cm below the surface of the seawater in the 1L glass flasks, that contained 100 mL of air as headspace. After adding the sediment, seawater, and substrates to the flasks O_2_ measurements were made over time during the incubations using a Fibox connected to a fiber-optic cable (PreSens), using 4 °C as the calibration temperature which was the temperature of the incubations. All O_2_ concentrations were measured in µM (S2 Fig) over time in the controls and substrate addition incubations from both the seawater and sediment incubations.

### DNA extraction, qPCR, and biomass estimation

We extracted DNA from sediment and water samples and incubations following established protocols [15]. In brief, DNA from sediments was extracted using a sodium phosphate extraction buffer and bead beating, followed by centrifugation and concentration of the supernatant containing the DNA in 30 KDa amicon filters [15]. The concentrated supernatant was purified using the DNeasy PowerClean Pro Cleanup Kit (Qiagen). DNA from water samples (filters) was extracted using a sucrose buffer and bead beating, followed by an overnight proteinase K treatment and concentration of the supernatant in 30 KDa amicon filters [15]. The concentrate was purified using the DNeasy Blood & Tissue kit (Qiagen). Using these DNA extracts, qPCR of fungal 18S rRNA gene and bacterial/archaeal 16S rRNA genes was conducted in a CFX Connect real-time PCR system (Bio-Rad, Hercules, CA, USA) as described previously [37]. In brief, all qPCR reactions were prepared with the epMotion 5070 robotic pipetting system (Eppendorf) to ensure consistency in white 96-well plates (BioRad) [37]. As described previously, quantification of prokaryotic 16S rRNA genes was done using the primer pair 515F/806R [37], and fungal 18S rRNA genes were quantified using fungal-specific primers FR1 (5′-AICCATTCAATCGGTAIT-3′) and FF390 (5′-CGATAACGAACGAGACCT-3′) [76]. Reactions were performed in a CFX Connect real-time PCR system (Bio-Rad, Hercules, CA, USA). No-template controls were added to assess contamination during the process. In order to reduce the amplification of non-fungal eukaryote 18S rRNA genes (from protists, algae, etc.) that can occur in marine samples with the fungal FR1/FF390 primer pair, we used blocking primers designed to prevent non-fungal amplification [56] in all fungal 18S qPCR reactions. All statistical analyses of qPCR data were performed in R (4.3.1) with RStudio. Heatmap plot of fungal to bacteria qPCR abundance ratios (F:P ratios) across the fjord transect was visualized using Ocean Data View [57].

In order to more directly compare the biomass of fungi and prokaryotes (due to the differences in rRNA gene copy numbers and C content per cell), we converted the fungal 18S rRNA gene and prokaryotic 16S rRNA gene abundances determined via qPCR into biomass estimates. For fungi, the fungal-specific 18S rRNA gene copy abundance was converted into fungal biomass C using the empirically determined conversion factors of 0.264 pg C per fungal ITS1 copy for soil fungi [39] and 0.12 pg C per fungal 18S copy established for marine fungi in sediments [38]. For the terrestrial samples in our study, we converted the fungal 18S abundance into fungal biomass C using the soil conversion factor [39]. The fungal 18S from marine samples were converted into fungal biomass C using the conversion factor from marine sediments [38]. Prokaryotic 16S rRNA qPCR abundance was converted into biomass C using the conversion factor of 0.195 pg C per 16S rRNA gene copy for soil prokaryotes, that was empirically determined [40]. We acknowledge that these conversions from qPCR data are estimates, but should be more representative than a direct comparison of the qPCR data alone considering the large differences in rRNA gene copy numbers between fungi and bacteria. The positive correlation observed in the gene copy abundance between fungi and prokaryotes was mirrored after converting gene copies to fungal and prokaryotic biomass C using the established conversion factors (S1 Fig).

### Fungal ITS sequencing and analysis

The fungal ITS region was used for fungal barcoding as it is conventionally accepted as a suitable marker for fungal taxonomy [77]. The fungal ITS1 region was PCR amplified with the primer pair ITS1-F/ITS2 (ITS1-F: 5′-CTTGGTCATTTAGAGGAAGTAA-3′, ITS2: 5′-GCTGCGTTCTTCATCGATGC-3′) that also contained Illumina adapters and a unique barcode sequence for sample demultiplexing as described previously [37,78]. The ITS1-F/ITS2 PCR protocol consisted of three steps: (i) denaturation at 94 °C for 45 s, (ii) annealing at 51 °C for 30 s, and (iii) elongation at 72 °C for 90 s. The PCR was carried out for 40 cycles on a real-time qPCR machine (CFX-Connect BioRad) together with no-template controls on every PCR reaction using the same conditions as above. Using a real-time PCR machine provides additional sensitivity towards contamination because very small amounts of amplification can be observed after 35 cycles in the negative control that may not be visible on a gel. Real-time PCR runs that exhibited amplification in negative controls were not used for sequencing. The barcoded PCR protocol for fungal ITS1 used has been calibrated using a mock community of fungi, that was shown to have 80%–90% accuracy in capturing fungal richness [78]. PCR products were purified using QIAquick Gel Extraction Kit (Qiagen) as described and measured in a Qubit 3.0 fluorometer. Barcoded 16S rRNA and ITS1 amplicons were pooled at 1 nM and sequenced on the MiniSeq platform (Illumina).

For ITS1 quality control and OTU clustering of ITS1 sequences was performed using USEARCH (version 10) [79] with 97% sequence identity. For this, we used the same parameters as performed with a mock community that we determined previously to be 80%–90% accurate in capturing fungal OTU richness [78]. Namely, only reads were kept in the analysis that had ≤1 error (-fastq_maxee 1.0). After OTU clustering, only OTUs were only considered that had more than 10 sequences, lower abundance OTUs containing <10 reads were removed. The ITS taxonomy was assigned by BLASTn using the default parameters (word size = 28, gap open = 0, gap extend = 2.5, *E*-value < 10), of fungal ITS OTUs against the UNITE database (release 8) [80] which included non-fungal eukaryotes (protists). The taxonomy was assigned as the top BLASTn hit, and only hits with >90% nucleotide identity across 150 bp alignment were considered. Non-fungal OTUs were identified on the basis of their top BLASTn hits and removed from the final analysis. Ordination (NMDS) and Analysis of Similarity were performed in R using the Vegan package (https://CRAN.R-project.org/package=vegan). Analysis of Similarity was conducted using 999 permutations and Bray–Curtis dissimilarity.

### Ultracentrifugation and density gradient fractionation

Extracted DNA was prepared and processed for density gradient centrifugation following a previously established qSIP protocol [37]. Technical replicates were performed for selected samples, to assess the variability in ultracentrifugation and density gradient fractionation on the qSIP method (S2 Table). Moreover, biological SIP incubations on selected sites provided an assessment of the variability in microbial amino acid assimilation between replicate incubations (S2 Table). The EAF of prokaryotic 16S rRNA genes and fungal 18S rRNA genes from the labeled amino acid SIP incubations were calculated as previously described [37].

### Estimating excess atom fraction of total 16S and fungal 18S rRNA genes

The EAF was calculated as a measure of the amount of substrate incorporated into the DNA of either bacteria or fungi from the labeled amino acids. The EAF of bacterial 16S rRNA genes and fungal 18S rRNA genes from the labeled amino acid SIP incubations was calculated as previously described [37]. The EAF value should reflect the proportion of labeled atoms that are assimilated into the rRNA genes. For example, an EAF value of 0.2 would relate to 20% of stable isotope replacement.

Fungal:prokaryote EAF ratios were calculated using the median EAF of multiple technical replicates at each sampling site. Null results (no detectable labeling) were interpreted as amino acid assimilation being below our EAF detection limit of 0.01 (1% isotope replacement) [37]. Setting null results to an EAF of 0.01, allowed us to calculate a F:P EAF ratio in cases of no detectable amino acid assimilation.

### qSIP calculations

In order to quantify FAA assimilation by specific fungal taxa, SIP incubations showing the highest ^13^C EAF values for fungal 18S rRNA genes (S3 and S4 Figs) were selected for fungal ITS1 barcoding of the individual density fractions (resulting from density gradient ultracentrifugation). The sample that was chosen for fungal-specific qSIP was the site AWI2 because it had a relatively high amount of FAA assimilation as evidenced by the EAF of the fungal 18S rRNA genes (S3 and S4 Figs) and low ^13^CO_2_ remineralization rate. Three technical replicates of the density gradient fractionation were performed to quantify the variability within the sample. We performed barcoded ITS sequencing of 10 density fractions across three technical replicates of the control and ^13^C,^15^N-labeled SIP incubations (*n* = 60 total individual density fractions sequenced) from the inner fjord sediment site (AWI2) (S4 and S5 Figs).

We used the HTSSIP package [81] to calculate the ^13^C,^15^N-EAF for each individual OTU in the unlabeled incubations relative to the ^13^C,^15^N-labeled incubations across three technical replicates, and calculated confidence intervals using bootstrapping (999 bootstraps). From each incubation fractionation that consisted of 20 total fractions, the 10 density fractions containing the DNA peaks were selected for ITS sequencing following the protocol described above. The cutoff criteria for OTUs to be considered were (i) OTUs needed to be detected in at least two of the three technical replicates, (ii) a minimum of five ITS1 sequences per replicate needed to be detected, and (iii) the OTU needed to have been detected in at least two density fractions per replicate.

The amount of amino acid ^13^C or ^15^N assimilated by specific fungal taxa was calculated based on an isotopic replacement approach which consists of comparing the buoyant density of DNA for a specific OTU in the treatment with labeled amino acids against a control [52], as described previously for fungi [37]. In brief, we normalized the fractional sequence abundance of fungal ITS1 OTU sequences using the qPCR-determined abundance of fungal 18S rRNA genes across the density gradient fractions. For the fungal qPCR, we used 18S rRNA gene primers specific to Fungi as opposed to ITS1—because the large length variations in fungal ITS sequences hinders its usage for accurate qPCR quantification. However, the fungal 18S rRNA gene is much more conserved in length by comparison and permits a qPCR quantification [37].

We acknowledge that rRNA operon copy numbers vary between fungal taxa [59] and may bias fungal EAF comparison between different fungal taxa, especially in samples with very different fungal communities. However, this bias should be negligible when comparing EAF of the same fungal OTUs in different samples. Because the fungal rRNA operon is a continuous sequence of 18S, ITS1, 5.8S, ITS2, and 28S [82], any effects of copy number differences between the fungal 18S rRNA gene (used for the qPCR) and ITS1 (used for diversity barcoding) sequences should be negligible on these calculations [37]. The quantitatively normalized fungal ITS1 distributions were then used together with CsCl densities of each fraction to calculate the average buoyant density of each fungal OTU using the HTSSIP package for analyzing qSIP data [81].

Because we used ^13^C,^15^N-labeled amino acids as substrate to trace activity, the incorporation of ^15^N into the DNA, and its effect on the molecular weight, had to be accounted for in addition to the ^13^C contribution. We calculated separately the theoretical maximum molecular weight of an entirely ^15^N-labeled DNA following the equations in [51]:


$$\begin{array}{c} {\text{M}}_{\text{HEAVYMAX}i}\;=\;\text{0}.\text{5}\text{0}\text{2}\text{4}\text{8}\text{5}\text{1}\;\text{G}i\;+\;\text{3}.\text{5}\text{1}\text{7}\text{3}\text{9}\text{6}\;+\;{\text{M}}_{\text{LIGHT}i} \end{array}$$

where M_LIGHT*i*_ is the observed molecular weight of a single strand of DNA. To calculate the ^15^N enrichment for each taxonomical unit, we applied the following equation from [51] and implemented into HTSSIP [81]:


$$\begin{array}{c} A_{\text{NITROGEN}i}=\;\frac{M_{\text{LAB}i}-M_{\text{LIGHT}i}}{M_{\text{HEAVYMAX}i}-M_{\text{LIGHT}i}}\;.\;(\text{1}-\text{0}.\text{0}\text{0}\text{3}\text{6}\text{6}\text{3}\text{0}\text{0}\text{4}) \end{array}$$

The theoretical maximum molecular weight of fully ^13^C-labeled DNA for each taxon was calculated with the equations from Hungate and colleagues (2015) which is already included in the original code of the HTSSIP [81] package. To estimate the excess atom fraction (*A* values; EAF) caused by enrichment of ^13^C and ^15^N in the DNA of each taxon, we calculated ^15^N-EAF (*A*_NITROGEN_) under the assumption that entire density shift resulted from 100% ^15^N-labeling, while ^13^C-EAF values (*A*_CARBON_) were calculated assuming that the shift was exclusively due to ^13^C enrichment. We further assumed that microbial taxa assimilated these elements in the same relative proportions as provided in the amino acid mixture. Our mixture had a C:N atomic ratio of 3.78:1, corresponding to *f*(C) = 0.79 and *f*(N) = 0.21. To obtain a combined ^13^C + ^15^N-EAF for each OTU, the isotope-specific contributions were weighted according to these proportions using the following equation:


$$\begin{array}{c} A_{\text{COMBINED}}=\;fN*A_{\text{NITROGEN}i}+\;fC*A_{\text{CARBON}i} \end{array}$$

This approach accounts for the different proportions of C and N within DNA nucleotides, and respective potential contributions of ^13^C and ^15^N to increase the molecular weight of fungal 18S rRNA genes. The updated code to the HTSSIP pipeline, including the modifications we introduced to account for ^15^N contribution, is available from the authors upon request.

For the statistical analysis, we calculated the ^13^C and ^15^N isotopic enrichment, i.e., A_COMBINED_, between treatments and used bootstrapping resampling (*n* = 1,000) to estimate 90% confidence intervals of replicates within each treatment according to Hungate and colleagues (2015). This way, we estimated the total EAF per taxon as a result of the incorporation of ^13^C and ^15^N.

### Measuring remineralized ^13^C from SIP incubations

^13^CO_2_ produced during the SIP incubations was measured using GC–QMS as described previously [37,83]. Experimental treatments that had ^13^CO_2_ percentages higher compared to unlabeled controls (reflecting the natural abundance of ^13^C in the environment: 1.1% ± 0.05%), were indicative of respiration of the ^13^C-labeled amino acid substrates. The ^13^CO_2_ from SIP incubations was measured in biological and technical replicates from most incubations (S2 Table).

### Normalizing CO_2_ respiration to substrate concentration

Prior to comparing ^13^C remineralization from the ^13^C-labeled FAA SIP incubations in sediments and seawater, we therefore normalized the carbon remineralization against the added concentration of ^13^C-labeled FAAs. To this end, we first multiplied the %^13^C in the DIC (the remineralized FAAs, which was determined by the GC–QMS measurements described above) by the known concentrations of DIC reported previously for seawater (2 mM) [84], marine surface sediment (2.2 mM DIC) [85], GFSs (0.6 mM) [6], and tundra soil (1 mM) [86] from Svalbard ecosystems. This provided the µmoles of ^13^C per mL in the DIC pool, which could be divided by the added ^13^C-substrate concentration (in mg mL^−1^). The resulting equation therefore provides an estimate of the millimoles of ^13^CO_2_ produced per mg of substrate:


$$\begin{array}{c} \mu \text{moles }\text{13}{\text{CO}}_{\text{2}}\text{ per mg substrate }=\;[(\%\text{1}\text{3}\text{C in DIC})\;\times \;(\text{DIC }\mu \text{moles }{\text{mL}}^{-\text{1}})]/(\text{1}\text{3}\text{C}-\text{substrate mg }{\text{mL}}^{-\text{1}}) \end{array}$$

### Statistical analysis

We examined the relationship between fungal and prokaryotic biomass running a Spearman’s rank correlation test, and we also built a linear model with the log-transformed biomass variables to represent the relationship graphically in the plot in Fig 1B. Similarly, we measured the correlation between the qPCR abundance ratio (F:P ratio) and the normalized ^13^CO_2_ concentration, shown in Fig 4B. These statistical tests were performed in R (4.3.1) using RStudio. The two-sided Student *t* test used to compare the relative sequence abundance between fjord/GFS and soil samples for the fungal genera that assimilated FAAs primarily in the fjord sediments was performed in Excel.

## Supporting information

S1 Data An excel file that contains the numerical values used for the figures in the manuscript and supplemental figures (Figs 1B, 2A, 2B, 3A, 3B, 4A, 4B, 5, S1A, S1B, S1C, S1D, S2, S3, S4, S5, S6, S7). The file contains multiple worksheets that are numbered according to the figure number in the manuscript to which the values are derived.
(XLSX)

S1 Table Description of sample groupings and associated information. ML, Midtre Lovénbreen; AB, Austre Brøggerbreen; VB, Vestre Brøggerbreen; GFS, Glacier-Fed Stream.
(DOCX)

S2 Table Description of the Stable Isotope Probing (SIP) incubations setup and number of replicates in downstream analyses. GFS, glacier-fed stream; ML, Midtre Lovénbreen.
(DOCX)

S1 Fig **A:** Correlation, after converting gene copy abundances into biomass estimates for fungi and prokaryotes. **B:** Correlation of fungal (18S) and prokaryotic (16S) rRNA gene copy abundances across all samples. Estimates of fungal biomass from gene copy abundance were made using prior empirically established conversion factors for fungi from soil (https://doi.org/10.1007/s42832-024-0243-5) and marine sediment (https://doi.org/10.1016/j.pocean.2018.09.011). Estimates of prokaryotic biomass from gene copy abundance was made from were made using prior empirically established conversion factors for prokaryotes from soil (https://doi.org/10.1128/AEM.00441-08). **C:** Fungal prokaryote biomass ratios after conversion. See Methods for more details. **D:** Box plots of fungal to prokaryotic abundance based on qPCR gene abundance ratios. The data underlying this Figure can be found in S1 Data or at https://doi.org/10.5281/zenodo.19493690.
(EPS)

S2 Fig Concentration of dissolved O_2_ over time in SIP incubations from surface seawater (left) and sediment (right). Incubations were supplemented with either amino acids, cellulose, or no substrate added (control). The data underlying this Figure can be found in S1 Data or at https://doi.org/10.5281/zenodo.19493690.
(EPS)

S3 Fig Normalized concentration of ^13^CO_2_ (as percentage of ^12^CO_2_) after seven days of incubation. ^13^CO_2_ percent was normalized to the concentration of added ^13^C-labeled substrate in the incubations (sediments: 10 µg/mL, seawater 0.1 µg/mL). Vertical bars represent averages from three technical replicates, error bars represent standard error (see S2 Table for the number of technical replicates per biological replicate. Biological replicates are plotted individually, and indicated with “A,” “B,” “C.” Subsurface and surface sediment samples are indicated with “a” and “b,” respectively. ^13^C-substrate type is represented by full color (^13^C amino acids) or dashed lines (^13^C-cellulose). The horizontal dotted line is the detection limit for ^13^C-labeling in CO_2_. ML: Midtre Lovénbreen. Note that the y-axis is in logarithmic scale. The data underlying this Figure can be found in S1 Data or at https://doi.org/10.5281/zenodo.19493690.
(EPS)

S4 Fig DNA-SIP results from labeled (orange) and unlabeled (blue) amino acids in fjord sediments. Plots show density gradient fractionation of fungal 18S rRNA and 16S rRNA genes (determined via qPCR) in the controls (blue) and ^13^C-incubations (orange). Y axis: normalized abundance of rRNA genes across the CsCl gradient (% of maximum fraction value). Technical replicates are depicted with squares, circles, and triangle points. Horizontal bars show calculated excess atom fraction (EAF). Technical replicates are displayed as multiple bars within each biological replicate. See S2 Table for full reporting of technical and biological replicates for SIP incubations. The data underlying this Figure can be found in S1 Data or at https://doi.org/10.5281/zenodo.19493690.
(EPS)

S5 Fig DNA-SIP results from labeled (orange) and unlabeled (blue) amino acids in fjord seawater (2m water depth). Plots show density gradient fractionation of fungal 18S rRNA and 16S rRNA genes (determined via qPCR). The *y* axis shows the normalized abundance of rRNA genes across the CsCl gradient (% of maximum fraction value) in unlabeled controls (blue) and labeled incubations (orange). Technical replicates are depicted with squares, circles, and triangle points. Horizontal bars show calculated excess atom fraction (EAF) for each replicate. Technical replicates are displayed as multiple bars within each biological replicate. Labels “A” and “B” refer to biological replicates. See S2 Table for full reporting of SIP technical and biological replicates. The data underlying this Figure can be found in S1 Data or at https://doi.org/10.5281/zenodo.19493690.
(EPS)

S6 Fig DNA-SIP incubation results from ^13^C-labeled (orange) and unlabeled (blue) cellulose in sediments at the AWI2 site (see map in Fig 1). Plots show density gradient fractionation of fungal 18S rRNA and 16S rRNA genes (determined via qPCR). The *y* axis shows the normalized abundance of rRNA genes across the CsCl gradient (% of maximum fraction value) in unlabeled controls (blue) and ^13^C-incubations (orange). See S2 Table for full reporting of SIP technical and biological replicates. The data underlying this Figure can be found in S1 Data or at https://doi.org/10.5281/zenodo.19493690.
(EPS)

S7 Fig Relative abundance of ITS sequences affiliated with specific fungal genera across the Kongsfjorden catchment area (Fig 1), that were found to be primarily assimilating free amino acids in the fjord sediment qSIP experiments (as opposed to glacial till and tundra soil). Note that the relative abundance of these groups is primarily restricted to the fjord sediments and seawater, and have a similarly high relative abundance in the GFS. The data underlying this Figure can be found in S1 Data or at https://doi.org/10.5281/zenodo.19493690.
(EPS)

## Abbreviations

DIC: dissolved inorganic carbon
DOC: dissolved organic carbon
DOM: dissolved organic matter
DON: dissolved organic nitrogen
EAF: excess atom fraction
FAAs: free amino acids
GC–QMS: gas chromatography quadrupole mass spectrometry
GFSs: glacier-fed streams
ITS: internal transcribed spacer
OC: organic carbon
OTUs: operational taxonomic units
PES: polyethersulfone
POM: particulate organic matter
qPCR: quantitative PCR
qSIP: quantitative DNA stable isotope probing
rRNA: ribosomal RNA
