# Supplementary material for: Fungi enhance microbial carbon retention in high Arctic fjord sediment
Source: PLoS Biol. 2026 Jun 16;24(6):e3003783. doi: 10.1371/journal.pbio.3003783 (PMC13271452; doi:10.1371/journal.pbio.3003783)
Supplement: S1 Table — ML, Midtre Lovénbreen; AB, Austre Brøggerbreen; VB, Vestre Brøggerbreen; GFS, Glacier-Fed Stream. (DOCX) [file pbio.3003783.s002.docx]

**Table S1.** Description of the sample’s groupings and information associated with the sampling. ML: Midtre Lovénbreen; AB: Austre Brøggerbreen; VB: Vestre Brøggerbreen; GFS: Glacier-Fed Stream.

| **Group** | | **Sub-group** | **Year of collection** | **# Samples** | **F:P abundance mean (± se)** | **Habitat description** | **Sampling technique** |
| --- | --- | --- | --- | --- | --- | --- | --- |
| **Glacier** | | Glacier sediments ML | 2021+2023 | 6 | 0.086 (±0.025) | Supraglacial sediments or cryoconite | Spatula surface sample |
|  |  | Glacier water ML | 2023 | 1 | 0.133 | Supraglacial water channels | Water filtration |
|  |  | Glacier sediments AB | 2023 | 2 | 0.106 (±0.023) | Supraglacial sediments or cryoconite | Spatula surface sample |
|  |  | Glacier subsurface sediments AB | 2023 | 7 | 0.027 (±0.004) | Supraglacial sediments, vertical profile | Syringe push-core |
| **Snout** | | Snout sediments ML | 2021+2023 | 15 | 0.369 (±0.173) | Rock flour sediments | Spatula surface sample |
|  |  | Snout subsurface sediments ML | 2023 | 44 | 0.025 (±0.019) | Rock flour sediments, vertical profile | Syringe push-core |
|  |  | Snout sediments AB | 2023 | 12 | 0.110 (±0.020) | Rock flour sediments | Spatula surface sample |
|  |  | Snout subsurface sediments AB | 2023 | 29 | 0.001 (±0.0001) | Rock flour sediments, vertical profile | Syringe push-core |
|  |  | Snout sediments VB | 2023 | 12 | 0.024 (±0.012) | Rock flour sediments | Spatula surface sample |
| **Forefield** | **Soil Chronosequence** | Young soils | 2021 | 13 | 0.021 (±0.004) | Recently exposed soils | Spatula surface sample |
|  |  | Inner moraines | 2021 | 6 | 0.024 (±0.006) | Inner moraines soils | Spatula surface sample |
|  |  | Intermediate soils | 2021 | 8 | 0.013 (±0.003) | Intermediately developed soils | Spatula surface sample |
|  |  | Terminal moraine | 2021 | 4 | 0.017 (±0.003) | Terminal moraine soils | Spatula surface sample |
|  |  | Tundra soils | 2021 | 7 | 0.024 (±0.005) | Tundra, fully vegetated soils | Spatula surface sample |
|  | **GFS** | Glacial Fed Stream sediments | 2023 | 3 | 0.529 (±0.152) | Freshwater sediments | Spatula surface sample |
|  |  | Glacial Fed Stream water | 2023 | 3 | 0.885 (±0.208) | Freshwater | Water filtration |
| **Inner Fjord** | | Mixing zone sediments | 2023 | 8 | 0.532 (±0.112) | Brackish | Spatula surface sample |
|  |  | Mixing zone water | 2023 | 1 | 1.766 | Brackish | Water filtration |
|  |  | Marine sediments | 2023+2024 | 8 | 3.620 (±0.739) | Marine | van Veen grab and box coring |
|  |  | Seawater | 2023+2024 | 26 | 0.165 (±0.070) | Marine | Water filtration |
|  |  | Beach | 2021 | 6 | 0.001 (±0.0002) | Marine, coastal | Spatula surface sample |
| **Outer Fjord** | | Marine sediments | 2023+2024 | 4 | 0.839 (±0.491) | Marine | van Veen grab |
|  |  | Seawater | 2023+2024 | 15 | 0.010 (±0.004) | Marine | Water filtration |
