# Supplementary material for: Fungi enhance microbial carbon retention in high Arctic fjord sediment
Source: PLoS Biol. 2026 Jun 16;24(6):e3003783. doi: 10.1371/journal.pbio.3003783 (PMC13271452; doi:10.1371/journal.pbio.3003783)
Supplement: S2 Table — GFS, glacier-fed stream; ML, Midtre Lovénbreen. (DOCX) [file pbio.3003783.s003.docx]

**Table S2.** Description of the Stable Isotope Probing (SIP) incubations setup and number of replicates in downstream analyses. GFS: glacier-fed streams; ML: Midtre Lovénbreen.

| **Sample Group** | **Site** | **Material** | **Description** | **Incubation replicates** | **Substrate added** | **Fractionation technical replicates** | **GC-MS technical replicates** |
| --- | --- | --- | --- | --- | --- | --- | --- |
| Glacier | ML-cryo | sediment | Cryoconite sediment | ML-cryo | amino acids | 1 | 1 |
| Snout | ML-X5 | sediment | Glacier snout sediment | ML-X5A | amino acids | 0 | 2 |
|  |  |  |  | ML-X5B | amino acids | 0 | 2 |
|  |  |  |  | ML-X5C | amino acids | 0 | 2 |
| GFS | F2 | sediment | Glacial Fed Stream sediment | F2 | amino acids | 1 | 2 |
| Mixing Zone | 011 | subsurface sediment (10 cm deep) | Brackish anoxic sediment (1 m water depth) | 011a | amino acids | 1 | 2 |
|  |  | surface sediment | Brackish sediment (1 m water depth) | 011b | amino acids | 0 | 2 |
|  | 021 | subsurface sediment (10 cm deep) | Brackish anoxic sediment (1 m water depth) | 021a | amino acids | 1 | 2 |
|  |  | surface sediment | Brackish sediment (1 m water depth) | 021b | amino acids | 0 | 2 |
|  | 013 | sediment | Brackish sediment (2 m water depth) | 013 | amino acids | 3 | 2 |
|  |  | water | Brackish water (2 m water depth) | 013A | amino acids | 3 | 3 |
|  |  |  |  | 013B | amino acids | 1 | 3 |
|  |  |  |  | 013C | amino acids | 0 | 3 |
|  |  |  |  |  | cellulose | 0 | 3 |
|  | 014 | sediment | Brackish sediment (10 m water depth) | 014 | amino acids | 1 | 2 |
| Inner Fjord | AWI2 | sediment | Marine sediment (120 m water depth) | AWI2 | amino acids | 3 | 2 |
|  |  |  |  |  | cellulose | 0 | 1 |
|  | K4 | water | Fjord seawater (2 m water depth) | K4A | amino acids | 2 | 3 |
|  |  |  |  | K4B | amino acids | 0 | 3 |
|  |  |  |  | K4C | amino acids | 0 | 3 |
|  | K5 | water | Fjord seawater (2 m water depth) | K5A | amino acids | 2 | 3 |
|  |  |  |  | K5B | amino acids | 0 | 3 |
|  |  |  |  | K5C | amino acids | 0 | 3 |
| Outer Fjord | AWI1 | sediment | Marine sediment (330 m water depth) | AWI1 | amino acids | 2 | 2 |
|  |  |  |  |  | cellulose | 0 | 2 |
|  | K2 | water | Fjord seawater (2 m water depth) | K2A | amino acids | 3 | 3 |
|  |  |  |  | K2B | amino acids | 1 | 3 |
|  |  |  |  | K2C | amino acids | 0 | 3 |
